# The effects of a 12-week combined motor control exercise and isolated lumbar extension intervention on lumbar multifidus muscle stiffness in individuals with chronic low back pain

**DOI:** 10.3389/fphys.2024.1336544

**Published:** 2024-08-26

**Authors:** Audrey Tornblom, Neda Naghdi, Meaghan Rye, Chanelle Montpetit, Maryse Fortin

**Affiliations:** ^1^ Department of Health, Kinesiology and Applied Physiology, Concordia University, Montreal, QC, Canada; ^2^ School of Health, Concordia University, Montreal, QC, Canada

**Keywords:** low back pain, motor control exercise, lumbar multifidus muscle, isolated lumbar extension, shear wave elastography

## Abstract

**Introduction:**

Exercise therapy is the primary endorsed form of conservative treatment for chronic low back pain (LBP). However, there is still conflicting evidence on which exercise intervention is best. While motor control exercise can lead to morphological and functional improvements of lumbar multifidus muscle in individuals with chronic LBP, the effects of exercise prescription on multifidus stiffness assessed via shear wave elastography are still unknown. The primary aim of this study is to determine the effects of a combined motor control and isolated lumbar extension (MC + ILEX) intervention on lumbar multifidus muscle stiffness.

**Methods:**

A total of 25 participants aged 18 to 65 were recruited from local orthopedic clinics and the university community with moderate to severe non-specific chronic LBP. Participants performed a 12-week MC + ILEX intervention program. Stiffness of the lumbar multifidus muscle (primary outcome) at L4 and L5 was obtained at baseline, 6-week, and 12-week using shear wave elastography. Changes in stiffness ratio (e.g., ratio of lumbar multifidus muscle stiffness from rest to contracted) were also assessed at both time points. Pre to post-intervention changes in lumbar multifidus muscle stiffness were assessed using a one-way repeated measure ANOVA.

**Results:**

Following the 12-week intervention, there were no statistically significant changes in lumbar multifidus muscle stiffness at rest on the right side at L4 (*p* = 0.628) and the left side at L4 and L5 (*p* = 0.093, *p* = 0.203), but a statistically significant decrease was observed on the right side at L5 (*p* = 0.036). There was no change in lumbar multifidus muscle stiffness ratio.

**Conclusion:**

This study provides preliminary evidence to suggest that a 12-week MC + ILEX intervention had minimal effect on lumbar multifidus muscle stiffness in individuals with chronic LBP. Further investigations are needed to confirm our findings and clarify the relationship between muscle stiffness and functional outcomes.

## 1 Introduction

Low back pain (LBP) is a leading cause of disability worldwide, with up to 80% of the adult population experiencing it at some point in their lives (Meucci et al., 2015; Fortin et al., 2021; Maselli et al., 2020). LBP places a significant financial burden on the healthcare and economic system due to the costs associated with ongoing care and work absenteeism (Meucci et al., 2015; Fortin et al., 2021). Although the multifaceted nature of LBP is well recognized, there are few effective conservative management programs available (Hartvigsen et al., 2018). The lumbar multifidus muscle is crucial for maintaining both lumbar segmental stability and dynamic stability of the spine (Hildebrandt et al., 2017). Growing evidence suggests that individuals with chronic LBP frequently exhibit impairments in the lumbar multifidus muscle (Deodato et al., 2024). These impairments are characterized by morphological changes, including fatty infiltration (Stokes et al., 2007), decreased cross-sectional area (Hides et al., 2008), and asymmetries along with functional deficits including increased stiffness and decreased strength (Fortin et al., 2021; Hildebrandt et al., 2017; Deodato et al., 2024; Nandlall et al., 2020; Murillo et al., 2019). The degenerative changes in the lumbar multifidus muscle can significantly impair function, leading to reduced motor control, diminished force production and delayed muscle activation (Hildebrandt et al., 2017; Danneels et al., 2002; Ehsani et al., 2017; Taljanovic et al., 2017). Such impairments may contribute to the persistence and exacerbation of chronic LBP symptoms. Given such findings, there is a growing interest in using diverse imaging modalities to investigate and quantify mechanical, morphological, and functional characteristics of the paraspinal muscles and their potential associations with LBP disability and related spinal pathologies (Deodato et al., 2024; Hodges et al., 2021). Understanding these characteristics could guide the development of targeted interventions aimed at improving muscle health and reducing LBP symptoms.

Shear wave elastography is a reliable non-invasive imaging tool used in research to quantify the mechanical and elastic properties of tissues such as stiffness and elasticity (Murillo et al., 2019; Taljanovic et al., 2017). The imaging tool complements conventional ultrasound techniques by enhancing the initial assessment and ongoing monitoring of various musculoskeletal conditions (Taljanovic et al., 2017). In a recent study, Murillo et al. (2019) reported increased lumbar multifidus muscle stiffness (i.e., higher shear modulus values) in individuals with chronic LBP as compared to healthy asymtomatic controls. The stiffness was also associated with a deficit in activation/contraction during isometric trunk extension. Koppenhaver et al. (2018) also found that lumbar multifidus muscle stiffness at rest was greater in individuals with LBP and that stiffness measures during lumbar multifidus muscle contraction/activation were correlated with self-reported pain and disability levels, but not with physical exam findings. Given that shear wave elastography is a valid and reliable tool to assess the biomechanical and viscoelastic properties of skeletal muscle in healthy and pathological conditions it could effectively be implemented in clinic and research settings to evaluate the effects of different therapeutic interventions (Creze et al., 2019).

Conservative treatment for chronic LBP encompasses a variety of approaches, including manual therapy, exercise therapy, electrical modalities, and pharmacological interventions, among others. Exercise therapy is the most common form of conservative treatment for individuals with chronic LBP (Hayden et al., 2021; Searle et al., 2015; Pinto et al., 2021; Shahtahmassebi et al., 2014). It has been shown to effectively reduce pain, disability and depression while also addressing compensatory motor patterns associated with LBP (Searle et al., 2015; Airaksinen et al., 2006; Steele et al., 2015; Rainville et al., 2004; Gordon and Bloxham, 2016; van Middelkoop et al., 2010; Koes et al., 2006; Shnayderman and Katz-Leurer, 2013). Given the extensive evidence linking LBP to muscular alterations (i.e., atrophy, fatty infiltration, asymmetry) in the trunk and paraspinal muscles, many exercise therapies are designed to improve the activation and control of these muscles (Steele et al., 2015; van Middelkoop et al., 2010; Koes et al., 2006; Ranger et al., 2017; Cuellar et al., 2017; Prins et al., 2018). While a recent systematic review suggested that Pilates and McKenzie therapy may be superior to other forms of exercise to improve pain and function in individuals with chronic LBP, the effect of such interventions on paraspinal muscle health (e.g., morphology, composition and stiffness) warrants further attention (Hayden et al., 2021; Searle et al., 2015; Pinto et al., 2021; Shahtahmassebi et al., 2014). Evidence supports motor control exercise and resistance training to improve lumbar multifidus muscle morphology such as increasing cross-sectional area and thickness (Searle et al., 2015; Pinto et al., 2021; Shahtahmassebi et al., 2014; Thibault et al., 2022). However, there is limited research on the impact of motor control exercise on lumbar multifidus muscle stiffness in individuals with chronic low back pain, aside from a recent case report demonstrating postoperative rehabilitation improvements in pain, disability and muscle morphology following lumbar total disc replacement (Searle et al., 2015; Pinto et al., 2021; Shahtahmassebi et al., 2014; Thibault et al., 2022). The case report also found a general decrease in muscle multifidus muscle stiffness in the prone position, whereas standing measurements remained relatively constant or increased post-surgery.

Individuals with LBP exhibit higher lumbar multifidus muscle stiffness (i.e., higher shear wave elastography values) compared to healthy controls, likely due to increased intramuscular fat and muscle spasms, which can negatively affect muscle strength by resisting muscle fiber shortening during contractions (Thibault et al., 2022). However, it is unknown if motor control combined with isolated lumbar extension (MC + ILEX) can modulate lumbar multifidus muscle stiffness/elasticity in individuals with chronic LBP. Therefore, the objective of this study was to assess the effects of a 12-week MC + ILEX intervention on lumbar multifidus muscle stiffness at L4 and L5 levels in individuals with low back pain (LBP). We hypothesized that a significant decrease in lumbar multifidus muscle stiffness at L4 and L5 levels would be observed post-intervention.

## 2 Methods

### 2.1 Study design and setting

This prospective intervention study was part of a larger two-arm randomized control trial (RCT) with a test-retest design, however, only data from the motor control and isolated lumbar extension (MC + ILEX) group was included in the current study. The larger RCT protocol has been previously published (Fortin et al., 2021), and the trial was prospectively registered (NTCT04257253). The larger RCT is now completed (Fortin et al., 2023), and all authors have authorized the data extrapolation for this study. All research activities were conducted at the School of Health, Concordia University. This study was approved by the Central Ethics Research Committee overseen by the Quebec Minister of Health and Social Services (#CCER-19-20-09). Each participant provided their informed consent by signing a consent form. This study was reported following the CONSORT guidelines (Schulz et al., 2010).

### 2.2 Participants

Individuals were eligible to participate in this study, provided they met all of the following inclusion criteria: 1) nonspecific chronic low back pain (LBP) for a minimum of 3 months (with or without accompanying leg pain), 2) were aged between 18 and 65 years old, 3) spoke in either English or French, 4) were seeking LBP care, 5) scored “moderate” or “severe” on the modified Oswestry Low Back Pain Disability Questionnaire, 6) not engaged in any physical activity or training targeting the lower back muscles within 3 months before the trial commenced (i.e., can be seen by a healthcare professional if core-specific exercises were not completed). Individuals were excluded if they met any of the following exclusion criteria: 1) sign of nerve root compression or motor reflex deficits; 2) history of spinal surgery, lumbar steroid injections, or vertebral fractures; 3) significant structural abnormalities in the lumbar spine (e.g., spondylosis, spondylolisthesis, scoliosis >10°); 4) pregnancy; 5) comorbidities that hinder safe participation in physical exercise, as determined by the Physical Activity Readiness Questionnaire. A physical examination was conducted by a Certified Athletic Therapist to confirm participants’ eligibility, if necessary (e.g., rule out neurological involvement) (Maselli et al., 2022).

A total of 25 participants (20 female, five male) were included in this study and selected from the larger RCT. Only participants enrolled in the MC + ILEX group were included in the current study. An *a priori* sample size calculation for the larger RCT was established based on the effect size (significant pre–post-difference in cross-sectional area measurements of the lumbar multifidus muscle following a motor control intervention) obtained from a prior study (Hides et al., 2012). G*power software (version 3.1) was used to calculate the sample size based on a power of 80%, a mean effect size of d = 0.90, a significance level of alpha 0.05, and a 10% buffer for potential loss to follow-up and 10% treatment non-adherence (Fortin et al., 2023).

### 2.3 Procedures

Participants underwent a 12-week intervention program involving two supervised exercise sessions weekly, each lasting approximately 45 min. The intervention was delivered by a certified athletic therapist with 1 year of experience. Throughout the intervention period, participants were asked to avoid seeking other forms of treatment (e.g., massage therapy, osteopath, chiropractor) and medication, although this did not hinder participation. Participants were asked to report any cointerventions at the end of the trial. Participants The participants completed a demographic questionnaire and questionnaires regarding their LBP history, pain, and disability during their first visit.

### 2.4 Ultrasound imaging protocol

**FIGURE 1:**
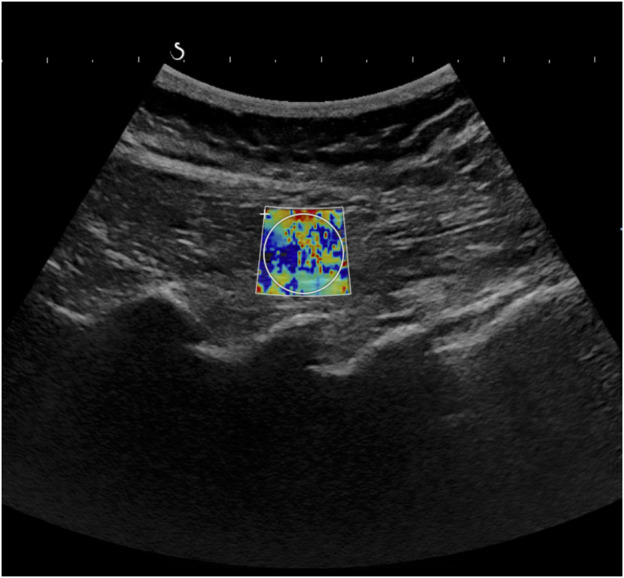
Shear wave elastography.

Ultrasound measurements were acquired at baseline, 6 weeks, and 12 weeks at the School of Health using the Aixplorer ultrasound unit combined with shear wave elastography. The lumbar multifidus muscle shear elastic modulus (measure for muscle stiffness in kPa) at L4 and L5 and levels was measured using an SL10-2 curvilinear ultrasound transducer with a 5 MHz frequency. The measurements were taken on the left and right sides both at rest and during submaximal contraction. Each image received two types of waves from the Aixplorer Multiwave: a compression wave which created a high-quality B-mode image and a shear wave that travelled through the tissue. The combination of these two waves on the image permits the shear wave modulus to be calculated and results in a quantitative colour-coded map of tissue stiffness (Figure 1).

### 2.5 Ultrasound lumbar multifidus muscle measurements at rest

The lumbar multifidus muscle measurement at rest was measured with the participants lying prone on the table with a pillow placed under their pelvis to decrease lumbar lordosis and maximize the contact between the transducer and the tissue. Before starting the measurement, the spinous processes (L4, L5 and S1) were identified through palpation and marked as a reference point. The ultrasound head was positioned around 2 cm lateral to the level of the lumbar spinous process in the sagittal plane. From this position, the ultrasound head was rotated 10° counterclockwise towards the frontal plane. The ultrasound head was then tilted by 10° from the sagittal plane to ensure that the ultrasound head was positioned medially towards the facet joint of the targeted spinous process. This position ensured that the transducer was placed approximately parallel to the lumbar multifidus muscle fibers. During the measurements, the clinician applied minimal pressure on the ultrasound probe to ensure it did not affect the shear wave elastography measurements. The shear wave elastography measurements were taken three times on the right and left sides at all levels.

### 2.6 Ultrasound lumbar multifidus muscle measurements during submaximal contraction

Participants were lying prone on the therapy table, with their elbows flexed to 90°, shoulders abducted to 120° and externally rotated to 90°. The ultrasound probe was placed in the same position as at rest. The submaximal contraction involved instructing the participants to perform a contralateral lift 5 cm above the table using a hand-held weight based on the participant’s body mass (Fortin et al., 2021; Naghdi et al., 2021). All contractions were held for 3–5 s with a minimum 30-s break between each contraction. Shear wave elastography measurements during submaximal contraction were taken 3 times per side at each spinal level and the mean was used in the statistical analysis. The shear elastic modulus means of each participant were divided by three (Koppenhaver et al., 2022).

All ultrasound images were downloaded onto a computer and transferred to the HOROS software for imaging analysis. The examiner analyzing the images was blinded to the participants’ demographic information, including age, gender, and any clinical history. To ensure objectivity, the images were coded with random identifiers.

### 2.7 MC + ILEX exercise intervention

The MC + ILEX intervention was split into two: the cognitive phase (Phase 1) and functional movements combined with ILEX (Phase 2). The motor control exercises covered the fundamental basics of muscle activation and breathing patterns, specifically addressing the identified deficiencies found during the assessment. The goal of the first phase was to decrease the activity of the global muscles and increase the activity of the deep trunk muscles. The starting positions for each exercise were progressed based on the abilities of the participants. Before moving to the second phase, participants were required to complete 10 repetitions while holding for 10 s, with minimal feedback or cues, and maintain a normal breathing pattern throughout the exercise. Both phases included diaphragmatic breathing that was incorporated into the exercises. In the second phase, the exercises were progressed towards functional activities. The exercises were performed while maintaining proper lumbar positioning and coordination of the deep trunk muscles consistently. The goal of the second phase was to automate the activation of the deep trunk muscles while coordinating the activation of the superficial muscles.

**FIGURE 2:**
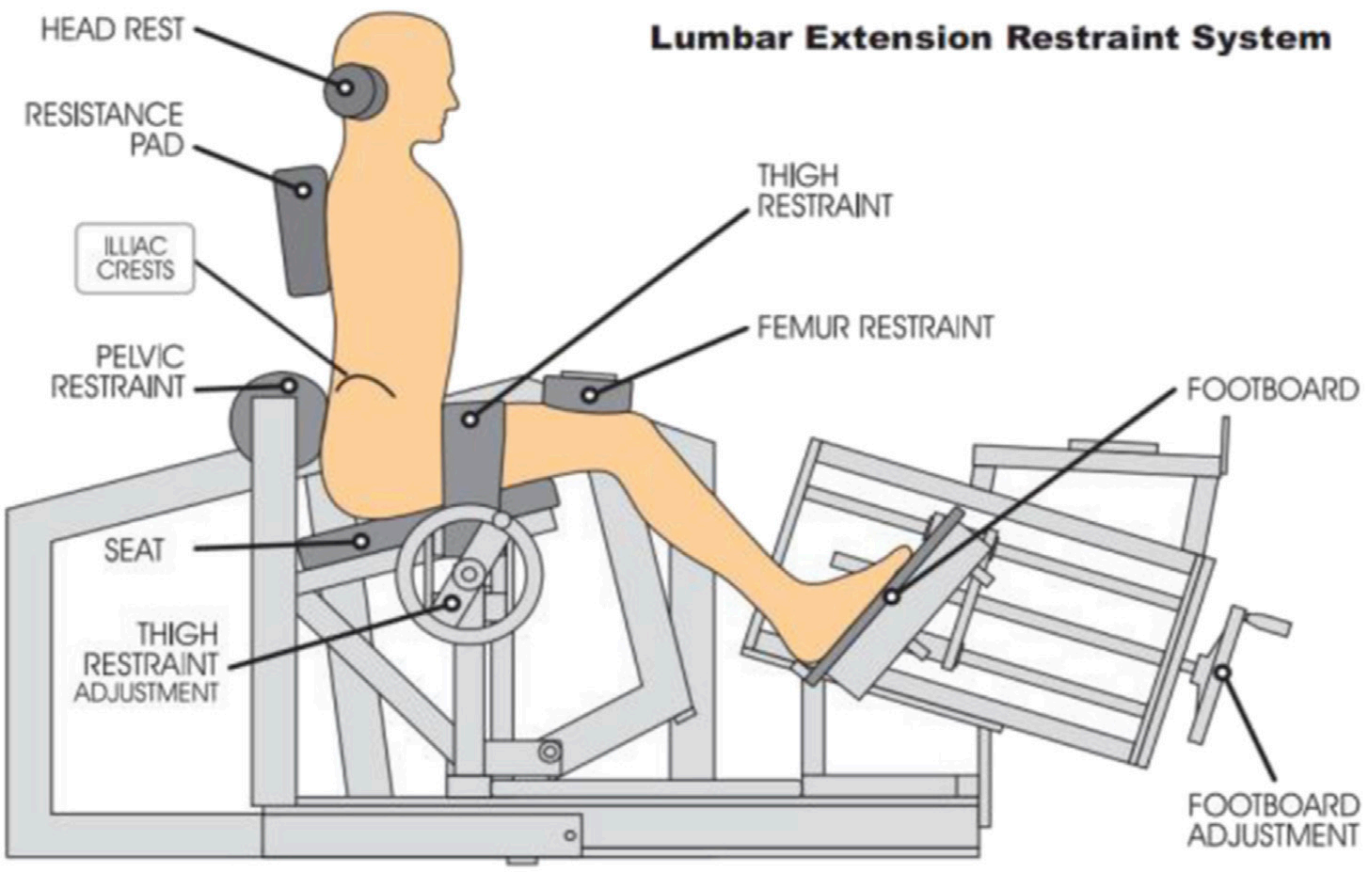
Schematic illustration of the MedX lumber medical machine.

The participants completed ILEX along with the motor control exercises. The ILEX (Figure 2) was completed on the MedX machine. The participants’ 1 RM was measured at baseline. To start, the participants completed two sets of 15–20 repetitions at 55% of their baseline 1 RM at 24°. Once the patients were able to complete 15-20 repetitions, they progressed by increasing the load by 5%. Refer to another study authored by Fortin et al., 2021 for a more comprehensive description of the completed intervention (Fortin et al., 2021).

### 2.8 Statistical analysis

Baseline characteristics were evaluated using descriptive statistics. Pre to post-intervention changes in lumbar multifidus muscle stiffness at rest and during contraction (e.g., lumbar multifidus muscle stiffness ratio) were assessed. The lumbar multifidus muscle stiffness ratio (%) for each side and spinal level was calculated using the following formula:

$$Stiffness\;ratio\;\left(\%\right)=\frac{Lumbar\;multifidus\;muscle\;stiffness\;rest}{Lumbar\;multifidus\;muscle\;stiffness\;contracted}$$

Pre to post-intervention changes in lumbar multifidus muscle stiffness at rest and stiffness ratio were assessed using one-way repeated measure ANOVA using “time” as the within factor. A separate analysis was performed for each side and spinal level. All statistical analyses were performed using IBM SPSS (version 28.0.0.0(190). New York, NY, USA); a *p*-value of <0.05 was considered statistically significant.

## 3 Results

### 3.1 Demographics

In total, 25 participants were enrolled in the MC + ILEX intervention and each of them successfully completed the 12-week intervention (no dropouts). The mean age of the participants was 45.16 years old (range 26–61 years old) and 20 (80%) were females (Refer to Table 1). Characteristics, clinical signs, symptoms, demographics, and questionnaire scores are presented in Table 1. Lumbar multifidus muscle stiffness measurements before and after the intervention are presented in Table 2. The mean stiffness measurement values of the 25 participants at both levels (L4 and L5) at rest and contracted are presented in Table 2. The lumbar multifidus muscle stiffness ratio values at L4 and L5 are presented in Table 3.

**TABLE 1:** Demographic characteristics of participants (n = 25).

Demographic characteristics	Mean (SD) or frequency (%)
Sex Male Female	20% 80%
Age (years)	45.16 ± 10.66
Height (cm)	169.68 ± 10.92
Weight (kg)	75.08 ± 16.39
BMI (kg/m^2^)	26.08
LBP duration (months)	73.52 ± 82.81
ODI Scores Baseline 6 weeks 12 weeks	29.40 22.96 19.08

LBP, low back pain; ODI, oswestry low back disability questionnaire; BMI, body mass index; BMI, units = kg/m^2^.

SD, standard deviation.

**TABLE 2:** One-way repeated measures ANOVA for lumbar multifidus muscle stiffness at rest.

Variables	Baseline (mean ± SD)	6 weeks (mean ± SD)	12 weeks (mean ± SD)	p-value
L4 Right Left	4.35 ± 2.08 4.06 ± 1.52	4.41 ± 2.09 4.55 ± 2.05	4.85 ± 2.21 4.19 ± 1.62	0.628 0.093
L5 Right Left	5.19 ± 2.40 3.83 ± 1.32	3.82 ± 1.57 4.55 ± 2.50	4.55 ± 2.13 3.84 ± 0.74	0.036 0.203

SD, standard deviation, *p-value < 0.05 statistically significant.

**TABLE 3:** One-way repeated measures ANOVA for lumbar multifidus muscle stiffness ratio.

Multifidus level	Baseline (mean ± SD)	6 weeks (mean ± SD)	12 weeks (mean ± SD)	p-value
L4 Right Left	0.32 ± 0.48 0.26 ± 0.30	0.33 ± 1.10 0.29 ± 0.54	0.35 ± 0.44 0.31 ± 0.48	0.792 0.133
L5 Right Left	0.36 ± 0.61 0.24 ± 0.35	0.28 ± 0.38 0.30 ± 0.37	0.35 ± 0.42 0.28 ± 0.38	0.372 0.339

SD, standard deviation, *p-value < 0.05 statistically significant.

### 3.2 Lumbar multifidus muscle stiffness at L4 and L5

**FIGURE 3:**
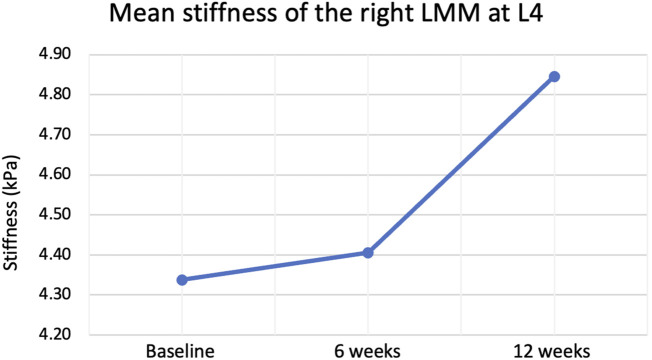
Mean stiffness of right lumbar multifidus muscle (LMM) at L4 at rest before, during and after the intervention.

**FIGURE 4:**
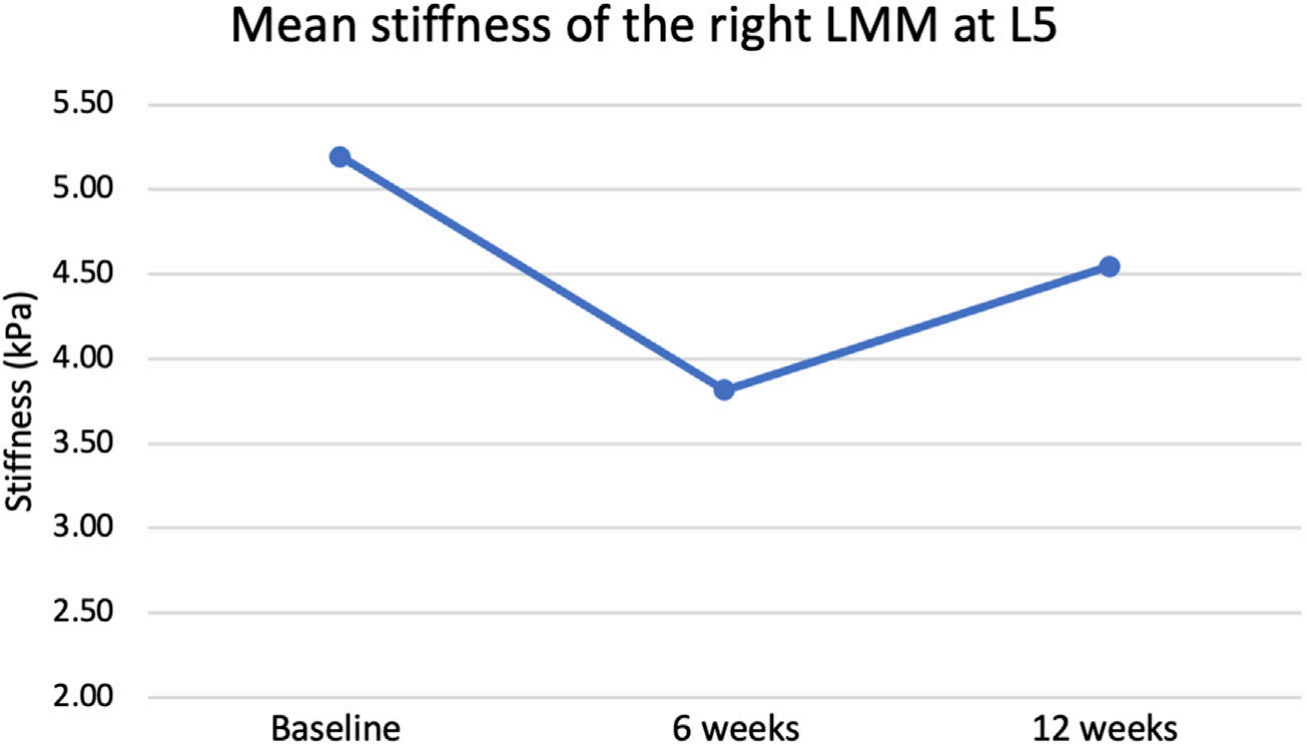
Mean stiffness of right lumbar multifidus muscle (LMM) at L5 at rest before, during and after the intervention.

The Greenhouse-Geisser correction revealed a non-statistically significant change in the right (*p* = 0.628) and left (*p* = 0.093) lumbar multifidus muscle stiffness at L4 (Refer to Table 2 and Figure 3). Similarly, there was no statistically significant change in lumbar multifidus muscle stiffness at L5 on the left side (*p* = 0.203) (Refer to Table 2; Figure 4). The Greenhouse-Geisser correction showed a statistically significant decrease in right lumbar multifidus muscle thickness at L5 (*p* = 0.036).

### 3.3 Lumbar multifidus muscle stiffness ratio

The Greenhouse-Geisser correction showed a non-statistical change in the lumbar multifidus muscle stiffness ratio at L4 on the right (*p* = 0.792) and left (*p* = 0.133) sides (Refer to Table 3). Similarly, the Greenhouse-Geisser correction showed a non-statistical change in the lumbar multifidus muscle stiffness ratio at L5 on the right (*p* = 0.372) and left (*p* = 0.339) sides (Refer to Table 3).

## 4 Discussion

### 4.1 Chronic low back pain and motor control exercise

Low back pain (LBP) represents a significant global health concern and cause of disability worldwide (Buchbinder et al., 2018). Amid increasing evidence revealing lumbar multifidus muscle dysfunction, including increased fatty infiltration, asymmetries, stiffness and decreased strength in individuals with chronic LBP (Fortin et al., 2021; Hildebrandt et al., 2017; Nandlall et al., 2020; Murillo et al., 2019), extensive research has aimed to discover interventions countering these morphological and functional changes. The dysfunction in the lumbar multifidus muscle leads to reduced motor control, diminished force production and delayed muscle activation (Hildebrandt et al., 2017; James et al., 2022). Exercise therapy serves as a potential way to counteract these dysfunctional changes. As such, recent systematic reviews demonstrated that Pilates, McKenzie therapy and functional reconditioning are considered the leading exercise forms to improve paraspinal muscle health (Hayden et al., 2021). Additionally, motor control exercise and resistance training are recommended to improve lumbar multifidus muscle morphology (Pinto et al., 2021; Shahtahmassebi et al., 2014). However, previous studies have mostly investigated the effects exercise on lumbar multifidus muscle morphology such as cross-sectional area and thickness (Pinto et al., 2021). This was the first study to investigate the effect of a 12-week intervention of MC + ILEX on the lumbar multifidus muscle stiffness at rest and contracted at L4 and L5 levels via shear wave elastography in participants with chronic LBP.

### 4.2 Lumbar multifidus muscle stiffness at rest

Inconsistent with our hypothesis, we did not find a decrease in lumbar multifidus muscle stiffness following the 12-week motor control and isolated lumbar extension (MC + ILEX) exercise intervention. This study revealed no significant change in resting lumbar multifidus muscle stiffness following the 12-week intervention program, except on the right side at L5, where a small decrease in resting lumbar multifidus muscle stiffness was observed. Previous studies showed that individuals with LBP have an increased lumbar multifidus muscle stiffness compared to healthy individuals (Murillo et al., 2019; Koppenhaver et al., 2018). Other studies have used cross-sectional area, thickness, and EMG to measure the effects of an exercise intervention on lumbar musculature in individuals with LBP. However, to our knowledge, this study is the first to use shear wave elastography to measure the effects of an exercise intervention on lumbar multifidus muscle stiffness. The lack of significant change in lumbar multifidus muscle stiffness may be attributed to an insufficient exercise volume, an aspect highlighted by Pinto et al., which they proposed as a crucial factor influencing the impact of motor control on lumbar multifidus muscle morphology (Pinto et al., 2021). Furthermore, Mannarino et al. conducted a study to determine the effects of resistance training on the patellar tendon stiffness (Mannarino et al., 2019). Participants performed an 8-week resistance training program for the quadriceps femoris muscles which included free-weight squats and knee extensions. Mannarino et al. found no detectable change in mechanical properties of the patellar tendon using shear wave elastography following the 8-week resistance training program (Mannarino et al., 2019). They suggested that the lack of effect on the patellar tendon stiffness may be attributed to the short intervention duration, aligning with the viewpoint expressed by Pinto et al., who indicated that an inadequate amount of exercise dosage might contribute to the minimal change observed in lumbar multifidus muscle stiffness while at rest (Pinto et al., 2021). Our study findings align with Akagi et al. (2015), who reported no change in the shear modulus of the triceps brachii muscle following a 6-week resistance training program. The lack of significant change in lumbar multifidus muscle stiffness in our study may also be due to the low exercise intensity (i.e., low load) and frequency (i.e., 2 times/week), which may not have been sufficient to induce changes in collagen content, collagen linking and tissue fluid (Buchbinder et al., 2018; Mannarino et al., 2019; Akagi et al., 2015). Additionally, we did not observe any changes in the percentage of fatty infiltration following the MC + ILEX intervention, as reported by Fortin et al. (2023). Although there was a significant increase in muscle cross-sectional area, the critical factor in altering muscle stiffness may hinge on improving muscle quality and composition rather than size alone. These findings suggest that targeted interventions, potentially involving higher intensity or frequency, are necessary to effectively alter muscle stiffness by improving muscle quality and composition.

### 4.3 Lumbar multifidus muscle stiffness ratio

The lumbar multifidus muscle stiffness ratio in individuals with chronic LBP defines the comparison between muscle stiffness at rest and during contraction. Prior research has often identified an alteration in this ratio, showcasing a difference in stiffness ratio between individuals with chronic LBP and those without chronic LBP (Murillo et al., 2019). Individuals with chronic LBP often exhibit a reduced ability of the lumbar multifidus muscle to effectively increase stiffness during muscle contraction compared to its resting state (Murillo et al., 2019). This reduction in the muscle’s ability to adequately stiffen when activated suggests a potential impairment in the muscle’s function, which could contribute to difficulties in providing spinal stability and movement support. The altered stiffness ratio in the lumbar multifidus muscle could contribute to difficulties in maintaining proper posture, executing coordinated movements, and providing essential spinal support during various activities. According to Murillo et al. (2019), individuals with chronic LBP exhibit a deficiency in contractile force due to a limited increase in muscular stiffness, potentially linked to the proliferation of collagen content or changes in connective tissue. However, our investigation did not reveal a significant change in the contractile ratio following the intervention, suggesting that the specific intervention had minimal impact on the lumbar multifidus muscle biomechanical and viscoelastic properties.

Presently, a universally recognized standard for the normal stiffness ratio in the lumbar multifidus muscle has not been established. Studies in the field primarily involve comparing stiffness ratios among various groups rather than establishing a definite benchmark for what constitutes a standard stiffness ratio in lumbar multifidus muscle. Therefore, future research should focus on differentiating between a typical stiffness ratio versus an altered one. Understanding the changes in the stiffness ratio of the lumbar multifidus muscle is essential for evaluating the functional limitations and developing targeted interventions to address the challenges individuals face due to chronic LBP.

### 4.4 Potential confounding variables

Potential confounding variables in this study include participant demographics such as age and sex, which may influence muscle stiffness and response to the motor control and isolated lumbar extension (MC + ILEX) exercise intervention (Viecelli and Ewald, 2022). Daily activities and lifestyle factors, such as occupational tasks, sleep, and stress levels, may impact muscle stiffness and overall intervention effectiveness (Stults-Kolehmainen and Sinha, 2014; Eijckelhof et al., 2013; Vinstrup et al., 2018). While operator variability and the timing of shear wave elastography assessments can introduce error, (Vaisvilaite et al., 2022), these factors remained consistent in our design. Furthermore, environmental factors like temperature and humidity may also influence shear wave elastrography measures, (Rasker et al., 1986), however, we suspect that this had minimal impact on our findings as all measurements were acquired in the same room (e.g., temperature remained constant).

### 4.5 Strengths and limitations

This study presented several strengths that contribute to its relevance in the field of chronic LBP management. First, the high adherence to the exercise intervention among participants underscores the feasibility and acceptability of the exercise intervention, ensuring that the observed outcomes are reflective of the intervention’s effects rather than non-compliance. Second, the study employed a valid and reliable outcome measure, shear wave elastography, a cutting-edge imaging modality known for assessing muscle stiffness, which adds depth to the analysis of the lumbar multifidus muscle. Additionally, the longitudinal design allows for a thorough analysis of the lumbar multifidus muscle changes throughout a 12-week exercise intervention. Nonetheless, there are some limitations including, a small sample size which may limit the generalizability of the findings, and the absence of a control group restricting the ability to establish a causal relationship between the intervention applied and lumbar multifidus muscle changes. Another limitation is the exclusive focus on lumbar multifidus muscle measurements taken at the L4 and L5 spinal levels, without the inclusion of the upper lumbar levels (i.e., L1-L3), which may not fully capture the effects across other spinal regions. Furthermore, the research did not stratify results by sex, potentially affecting the study’s applicability to different populations experiencing musculoskeletal dysfunction and pain.

### 4.6 Future research

Future research should aim to address potential confounding variables to gain a clearer understanding of the effects of exercise interventions on lumbar multifidus muscle stiffness in individuals with chronic LBP. A larger, more diverse sample size should be recruited to improve the generalizability of findings, and a control group should be included to compare the effects of various exercise interventions. Examining multiple lumbar spine levels (i.e., L1-S1) and stratifying participants by sex could provide more nuanced insights into how exercise interventions impact different subgroups. Exploring the impact of various exercise intensities and frequencies, as well as individual responses to motor control and isolated lumbar extension exercises, will provide deeper insights into the optimal exercise prescriptions for improving muscle stiffness and functional outcomes in patients with chronic LBP. Furthermore, future studies should incorporate longer follow-up periods to evaluate the sustainability of the intervention’s effect over time. This comprehensive approach will contribute to developing more effective, evidence-based conservative treatment programs for chronic LBP.

### 4.7 Clinical implications

The 12-week motor control and isolated lumbar extension (MC + ILEX) exercise intervention had minimal effect on lumbar multifidus muscle stiffness in patients with chronic LBP, indicating that while motor control and isolated lumbar extension exercises may improve muscle morphology (Fortin et al., 2023), it did not significantly alter muscle stiffness. This underscores the need for further research to confirm these findings and clarify the relationship between muscle stiffness and exercise interventions. Clinically, this suggests that a multifaceted approach to exercise therapy, tailored to individual patient needs and focusing on overall morphological and functional improvements and quality of life, may be beneficial.

## 5 Conclusion

The findings from this study suggest that a 12-week MC + ILEX intervention program had a minimal effect on lumbar multifidus muscle stiffness at L4 and L5 spinal levels. Further research should examine if different exercises with a longer intervention and higher loads can modulate lumbar multifidus muscle stiffness. While our study focused on the combination of motor control with isolated lumbar extension exercise on the lumbar multifidus muscle, it is important to note that chronic low back pain (LBP) is a multifaceted problem, which underscores the need for a comprehensive approach to understand and address the condition effectively. Our findings serve as a starting point for clinicians to expand upon and further investigate the effects of various exercises on lumbar multifidus muscle stiffness in individuals with chronic LBP.

## Data Availability

The original contributions presented in the study are included in the article/supplementary material, further inquiries can be directed to the corresponding author.

## Abbreviations

LBP, low back pain; MC + ILEX, motor control + isolated lumbar extension; SD, standard deviation; LMM, lumbar multifidus muscle.
